# Tomato fruit quality and nutrient dynamics under water deficit conditions: The influence of an organic fertilizer

**DOI:** 10.1371/journal.pone.0310916

**Published:** 2025-01-09

**Authors:** Maryam Zahedifar, Ali Akbar Moosavi, Edris Gavili, Arash Ershadi

**Affiliations:** 1 Department of Range and Watershed Management (Nature Engineering), Faculty of Agriculture, Fasa University, Fasa, IR Iran; 2 Department of Soil Science, College of Agriculture, Shiraz University, Shiraz, IR Iran; 3 Innovation Center of Zarnam Educators Research Industrial Group, Alborz Province, Hashtgerd City, Iran; Bahauddin Zakariya University Multan, Pakistan, PAKISTAN

## Abstract

Drought adversely affects the growth and performance of plants. By contrast, the application of organic modifiers can improve plant growth by supplying nutrients and water. The influence of foliar application of organic fertilizer under water deficit conditions on growth traits, chemical composition, and fruit quality of tomato (*Lycopersicon esculentum* Mill., var. Maya) were investigated in greenhouse conditions based on bi-plot and principal component analysis (PCA). Plants which were cultivated in soil under greenhouse conditions were subjected to four levels foliar spraying of Zargreen liquid organic fertilizer, ZLOF (0, 2.5, 5, and 7.5 L 1000^−1^, shown as Z0, Z2.5, Z5, and Z7.5, respectively), and three levels of soil water, SW (100, 75, and 50% of field capacity (FC), shown W100, W75, and W50, respectively). The results of biplot analysis using the different treatments representing 42.9% and 38.3%, 60.3% and 28.8%, and 63.1% and 22.4% of the variance attributed to the first two principal components (PCs) for the PC1 and PC2, under 100, 75, and 50% FC conditions, respectively. Water deficit induced a reduction of fruit dry, and fresh weights. Application of 2.5, 5, and 7.5 L 1000^−1^ of the organic fertilizer significantly increased fruit fresh weight by 16, 20, and 22% and fruit dry weight by 13, 20, and 20% as compared to that of control, respectively. Vitamin C content of fruit significantly increased by 16 and 33% when respectively 5 and 7.5 L 1000^−1^ of the organic fertilizer was foliar sprayed. Besides, fruit iron (Fe), sodium (Na (and potassium (K) concentrations increased with the application of the organic fertilizer at different levels of water deficit. Furthermore, the highest fruit zinc (Zn) concentration was obtained at the highest level of both applied organic fertilizer and water deficit. The best treatments were selected with increased PC1 and decreased PC2 for different water conditions. The W100Z7.5, W75Z7.5, and W50Z5 treatments with the higher PC1 and the lower PC2, also exhibited higher scores for fruit dry weight, and Na and K concentrations under W100; vitamin C, number of fruits, fruit fresh weight, and fruit Fe concentration under W75; citric acid, and fruit Fe, Zn, Na, K, and Cu concentrations under W50 treatment. The addition of the organic fertilizer was effective in enhancing the plant growth traits under water deficit conditions. Therefore, it can be concluded that organic fertilizer addition is an effective management strategy to mitigate the adverse effects of drought and improve the quantity and quality of tomato fruit.

## Introduction

Climate changes are being understood more and more due to the increasing tendency to urbanization, use of polluting fossil fuels, and incorrect managerial practices. Long-term droughts and water shortage crises in most parts of the world are the most important consequences. Osmotic changes, reduction of cell swelling, and cell wall elasticity are important responses of plants against water stress. Investigators reported that water stress as one of the biggest limitations of plant growth results in a decrease in the size of leaves, shoot dry weight, leaf area index, and compressive stress in plant tissues [1–5]. As a result, the plant’s physiological activities such as photosynthesis, respiration, absorption and transfer of nutrients and finally growth are reduced [6–8]. Under drought stress, on the one hand, limiting the absorption of nutrients and on the other hand, closing the stomata by the plant in order to reduce transpiration, prevent the entry of CO_2_, and reduce photosynthesis. Consequently, plant performance and development are negatively influenced [9, 10]. Furthermore, drought stress reduces the amount of chlorophyll and the relative humidity of the leaf, leading to the destruction of cell membranes and thus reducing photosynthesis [8, 11]. Since nutrient absorption and translocation processes such as mass flow closely depend on the amount of water; therefore, with a decrease in water, the absorption and transportation of nutrient elements decrease [12].

Under water stress conditions, the production of amino acids that are vital components in the plant is faced with problems; therefore, adding amino acids in the form of fertilizer mitigates the need for these compounds. As a result, the plant can use its stored energy to grow and increase the yield and quality of the product [13]. Bio-stimulants are biological and commercial organic products, a liquid mixture of natural plant compounds such as amino acids, glucosides, vitamins, micronutrients, growth hormones, or humic acids, which increase plant growth in tolerance to environmental stresses and improve the performance and quality of the products [14]. Organic fertilizers have a lot of nutritional value for crops, they increase the growth and yield of plants, and on the other hand, they play an effective role in preserving the environment and developing sustainable agriculture. Salehi et al. [15] stated that increasing soil fertility and plant production is the result of chelating essential elements and increasing their absorption. The increased permeability of the cell membrane to minerals is the reason for the increase in plant growth after adding organic compounds [16]. Sebastiano et al. [17] stated that organic compounds such as humic acid polymers, like adhesives, connect soil mineral particles and improve the soil structure, and penetration of water, air, and roots, as well as increase the activity of soil microorganisms. Bittelli et al. [18] showed that foliar application of organic compounds increased plant photosynthesis more than soil application, which could be due to better absorption of foliar-applied materials. Based on the findings of El-Bassiony et al. [19], humic acid foliar application increased the rate and amount of food absorption and as a result increased the amount of protein and chlorophyll in bean plants. Similar results were reported by Sabouri et al. [20] in *Satureja hortensis* L. plants.

Various mechanisms have been stated in order to increase plant resistance to water stress. Due to the presence of carboxylic groups and the continuous production of carbohydrates, organic compounds increase the water-holding capacity and improve the plant’s drought tolerance [21]. Organic acids such as humic acid can increase the absorption of water and nutrients from the soil by expanding plant rooting. Also, the role of these acids in reducing open pores and transpiration increases water absorption by the roots. The results of research by Hartz and Bottoms [22] showed that the formation of a network between humic acid molecules and soil micronutrients causes water storage, especially in light-textured soils. The bond between humic acid and water molecules will stabilize the soil temperature and prevent water evaporation. Results showed water stress significantly reduced the fresh weight of the oregano plant, while the application of humic acid increased it under the same conditions [23]. Tourfi and Shokuhfar [24] showed that the application of 300 mg L^-1^ humic acid increased wheat yield components and harvest index under drought stress at different growth stages. Sohag et al. [25] stated that the application of salicylic acid can activate the plant’s defense system and help the plant to adjust the water deficit conditions. This is due to the increased activity of antioxidant enzymes and the accumulation of active oxygen. According to predictions, water shortage will affect more than 50% of agricultural lands by 2050, and this is the biggest environmental threat that limits the growth of crops and vegetables.

Among vegetables, tomatoes are sensitive to water limitation and this harms the growth, production and quality of the fruit [26]. Tomato is one of the most important horticultural crops, and due to the abundance of mineral compounds, vitamins, and antioxidants in this plant, its cultivation is of interest in the world [27]. The limitation in tomato growth under water stress is attributed to the reduction of photosynthesis. This decrease is the result of slowing down the activity of ribulose 1, 5 bisphosphate carboxylase/oxygenase due to the limitation of the amount of CO_2_ in the intercellular space, which occurs as a result of stomata closure. As mentioned, tomato growth is affected by environmental factors such as water availability, so drought stress during the fruiting stage has reduced the yield by more than 17% [28]. The effect of liquid organic fertilizer on the growth, chemical composition, and quality of crops has not been investigated, especially in the conditions of water stress. In addition, considering the importance of tomato cultivation in the world, conducting research in order to increase the yield and quality of this product on the one hand and to minimize the negative effects of drought stress on the cultivation of this plant on the other hand, is of particular importance. It is hypothesized that foliar application of liquid organic fertilizers through supplying essential elements and compounds improves plant growth and affects the quantity and quality of the product. Furthermore, they may positively affect the growth of plants and reduce the adverse effects of drought under water stress conditions through regulation of the plant stomata. Since there is no research studying the effect of foliar application of organic liquid fertilizers on plant growth under water stress conditions. Therefore, the present research was conducted to investigate the effect of foliar spraying of Zargreen liquid organic fertilizer, which is an environmentally friendly byproduct of the Farhikhtegan Zarnam Research Industrial Group (Zar Green Refinery), on the growth characteristics, yield, fruit quality attributes, and chemical composition of the fruit of tomato plants under different water stress conditions based on bi-plot and PC analysis, and to select the best treatments concerning quantity and quality of the fruit. In other words, this is the first study to investigate the potential of Zargreen liquid organic fertilizer to mitigate the adverse effects of water deficit on tomato growth, yield, and its fruit quality and chemical composition.

## Materials and methods

### Preparation and analysis of the soil and organic fertilizer

The required amount of soil was collected from the depth of 0 to 30 cm of calcareous soil (silty clay) of Koye Asatid series, located in the Bajgah area of Fars province, the southwest of Iran (with an elevation of 1852 m above the mean sea level, 52° 46’ E longitude and 29° 50’ N latitude). The soil was air-dried and passed through a 2 mm sieve, and some physical and chemical properties were measured using the common standard methods (Table 1). Based on society’s need to produce health-oriented agricultural products with a focus on environmental protection, Farhikhtegan Zarnam Research Industrial Group (Zar Green Refinery) has produced a product called Zargreen liquid organic fertilizer (ZLOF). Liquid organic fertilizer is based on plant derivatives and is compatible with the environment. This product has a pH in the range of 3.5–4.5 and contains various amino acids in free form (6%), nutrients needed by plants including nitrogen (3%), phosphorus (2.5%), and potassium. (2%) as well as a high percentage of organic matter (30%) and organic carbon (11%).

**Table 1:** Selected characteristics of the soil along with their determination methods.

Property	Value (unit)	Reference
Soil texture class	Silty clay	[29]
pH of saturated paste	7.5	[30]
Organic matter, OM	1.1%	[31]
Cation exchange capacity, CEC	19 cmol_+_ kg^-1^	[32]
Calcium carbonate equivalent, CCE	42%	[33]
Electrical conductivity of saturated extract, EC	0.5 dS m^-1^	[34]
Available phosphorus, P	26 mg kg^-1^	[35]
Total nitrogen, N	0.1%	[36]
Available zinc, Zn	1.2 mg kg^-1^	[37]
Available manganese, Mn	4.3 mg kg^-1^	
Available iron, Fe	5.4 mg kg^-1^	
Available copper, Cu	0.1 mg kg^-1^	
Soluble sodium, Na	0.5 meq L^-1^	[38]
Soluble potassium, K	460 mg kg^-1^	
Soluble calcium, Ca	2 meq L^-1^	[39]
Soluble magnesium, Mg	1.3 meq L^-1^	

### Greenhouse experiment

A factorial experiment was conducted as a completely randomized design with three replications in greenhouse conditions from May 31, 2022, to September 26, 2022. Treatments consisted of four levels of foliar spraying of Zargreen liquid organic fertilizer, ZLOF (0, 2.5, 5, and 7.5 L 1000^−1^ shown as Z0, Z2.5, Z5, and Z7.5, respectively), and three levels of soil water, SW (100, 75, and 50% of field capacity (FC) shown as W100, W75, and W50, respectively). At first, 4 kg of the soil was placed in plastic bags. To prevent the probable nutrient deficiency; based on the results of the primary soil test, the required amount of nitrogen (N), iron (Fe), manganese (Mn), zinc (Zn), and copper (Cu) were added to soil as aquas solutions of urea, iron chelate (Fe-EDDHA), MnSO_4_, ZnSO_4_, and CuSO_4_, respectively. After that, the soil was mixed thoroughly and transferred into 4 kg cylindrical PVC pots. On May 31, 2022, eight tomato seeds (*Lycopersicon esculentum* Mill. var. Maya) were planted in each pot at the appropriate depth. After 4 weeks, the seedlings were thinned to two uniform plants per pot, and six weeks after planting to one plant per pot. After the establishment of the seedlings (two weeks after planting), the mentioned soil water levels were applied during the growing season by daily weighing the pots and compensating for the decreased water by adding the required amount of water. During the growing season, the minimum and maximum temperature of the greenhouse was measured with a thermometer. Three times, at 1.5, 2.5, and 3.0 months after planting, the mentioned liquid organic fertilizer solutions were foliar sprayed with the mentioned concentrations.

### Determination of growth parameters and chemical composition of plant

#### Plant harvesting, determination of fruit fresh and dry weight and nutrient contents

At crop maturity, the fruits were separated from the cut shoot of each plant, placed in paper bags, and counted for the number of fruits. After weighing (fresh weight) and washing with water and then with distilled water, were dried in an oven at 65°C, weighted, ground with an electric mill, and dry ashed at 550°C. The ashed samples were dissolved in 2 N HCl and passed through the Whatman 42 filter paper. The concentration of Ca, Mg, Fe, Mn, Cu, and Zn were determined by an atomic absorption spectrophotometer and Na and K were measured by a flame photometer.

#### Ascorbic acid (vitamin C) and titratable acids (citric acid) of fruit

To determine the content of ascorbic acid, fruit juices (0.1 mL) were extracted with 10 mL of 1% metaphosphoric acid. After the extracts were filtered, the filtrate (1 mL) was added to 9 mL of 50 μM 2, 6-dichloroindophenol (DIP), and the absorbance at 515 nm was measured with a T60 UV visible spectrophotometer [40]. Then the vitamin C content of the samples was determined using the corresponding standard calibration curve.

To measure the content of citric acid, two drops of phenolphthalein, as an indicator, were added to 5 ml of fruit juice and the sample was titrated with 0.1N NaOH until the pH reached 8.2. Then the consumption volume was recorded. The total amount of titratable acids was calculated using the following formula [41]:

$$\%Acid=\;\frac{\left(N\times V_{1}\times M.W.\right)}{\left(V_{2}\times 1000\right)}\;\times 100$$ (1)

where N and V_1_ are the normality and consumption volume of NaOH, respectively. V_2_ is the volume of the sample and M.W. is the molecular weight of the predominant acid (citric acid C_6_H_8_O_7_) which is equal to 192.124 g mol^-1^.

#### Statistical analysis of the data

Statistical analysis of the data was done using MSTATC statistical software. Two way ANOVA was performed to determine the effect of applied treatments on the studied attributes. Furthermore, the averages were compared with Duncan’s multiple range test at the statistical level of 5%. As well as Microsoft Excel and GraphPad Prism 9.0 programs were used for drawing diagrams. Furthermore, the principal component analysis (PCA) was applied to compare the treatments for all studied nutritional quality parameters of tomato fruit. Correlation analysis and PCA, based on the rank correlation matrix and biplot analysis were performed using the R-4.3.1 software packages.

## Results

### Effect of different levels of organic fertilizer and soil water on plant growth traits

#### Number of fruits

**Fig 1:**
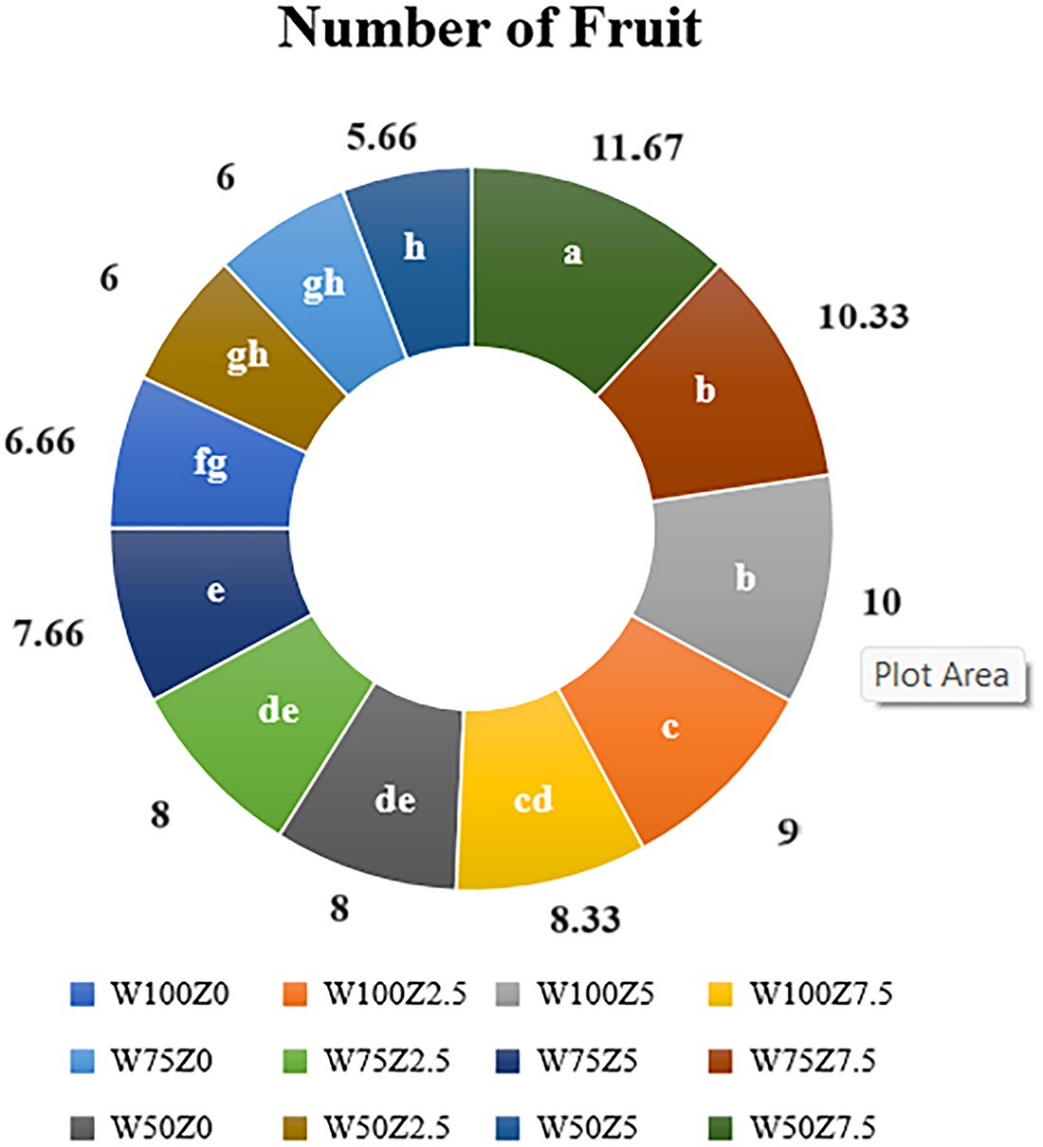
Effect of different levels of Zargreen liquid organic fertilizer (Z) and drought stress (W) on the average number of fruits per plant (Number of fruit).

The results of variance analysis showed that foliar application of ZLOF as well as SW had a significant effect on the number of fruits. The use of 75% and 50% FC compared to that of the control (100%FC, without drought stress) led to a decrease in the average number of fruits per plant by 6% and 8%, respectively (although only 8% reduction compared to that of control was significant). The results also showed that the application of 2.5, 5, and 7.5 L 1000^−1^ of ZLOF significantly increased the average number of fruits compared to that of the control by 11, 13, and 47%, respectively (Fig 1). The highest average number of fruits per plant was obtained when 7.5 L 1000^−1^ of the organic fertilizer was applied. As shown in Fig 1, W50Z7.5 indicated the maximum average number of fruits per plant (11.67) under a water deficit level of 50%FC, followed by W75Z7.5 (10.33) which corresponds to the 75%FC drought level with the same value of applied organic fertilizer (7.5 L 1000^−1^). According to the results, the application of the organic fertilizer at the maximum level (7.5 L 1000^−1^) decreased the negative impacts of water deficit on the number of fruits per plant.

#### Fruit fresh (FFW) and dry (FDW) weight

**Fig 2:**
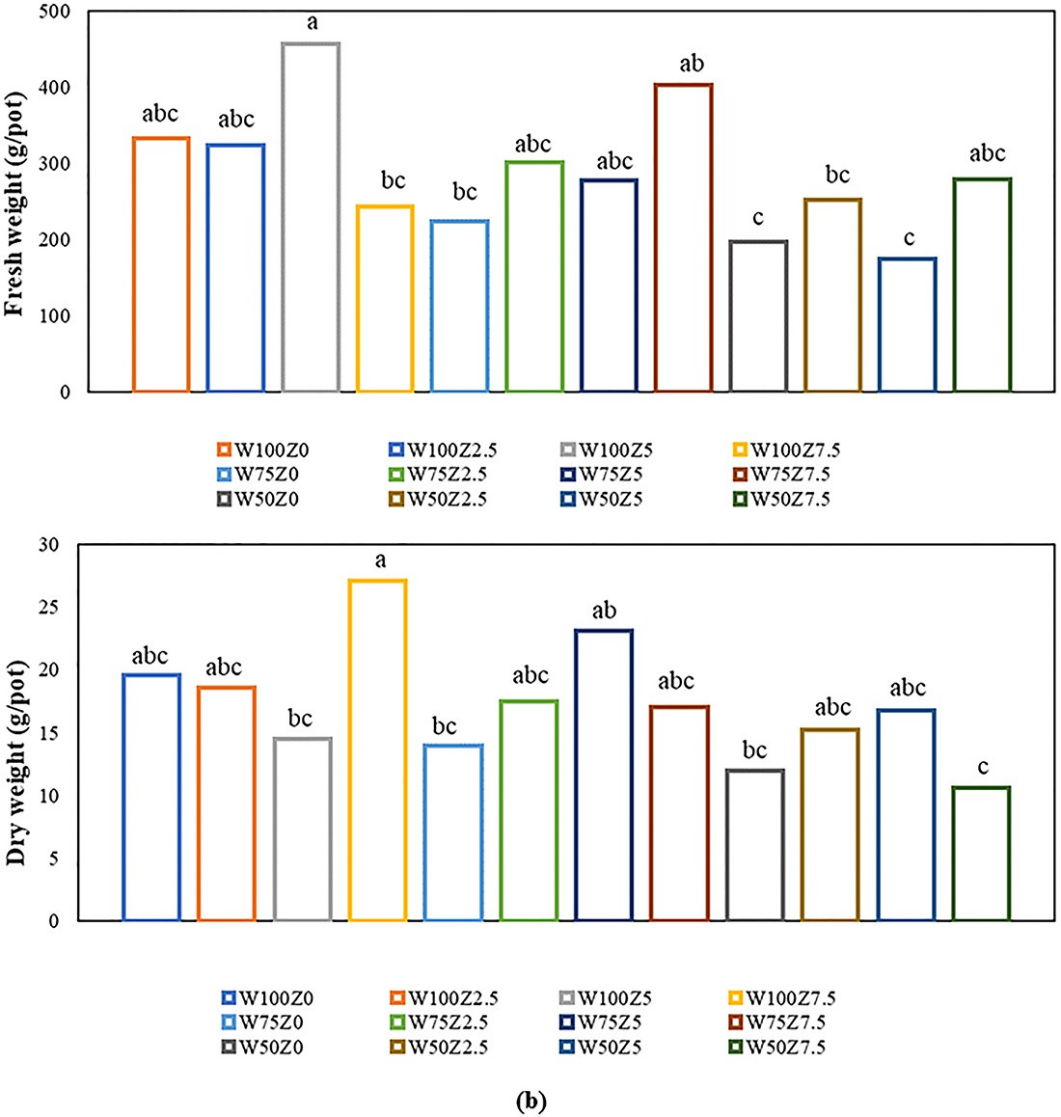
Effect of different levels of Zargreen liquid organic fertilizer (ZLOF) and drought stress (soil water, SW) on (a) fruit fresh (FFW) and (b) dry (FDW) weight of tomato.

Analysis of variance showed that both applied ZLOF and SW treatments had significant effects on the fruit fresh weight (FFW). The comparison of mean values also showed that FFW decreased by 11% and 34% at drought conditions of 75% and 50% FC as compared to that of control, respectively. The results also showed that the application of 2.5, 5, and 7.5 L 1000^−1^ of the studied organic fertilizer increased FFW significantly by 16, 20, and 22%as compared to that of the control, respectively (Fig 2a). The highest mean FFW (308 g pot^-1^) was obtained by adding 7.5 L 1000^−1^ of the organic fertilizer. Results showed that in the W100Z5 treatment, the FFW was the highest (456 g pot^-1^) among all of the experimental units. Treatments of W50Z0 (197 g pot^-1^), followed by W50Z5 (174 g pot^-1^) had the lowest FFW. While at the same level of water deficit (SW), the application of 7.5 L 1000^−1^ of the organic fertilizer increased it to 278 g pot^-1^.

The results of variance analysis showed that both foliar spraying of the organic fertilizer and soil water had a significant effect on the fruit dry weight. The comparison of mean values also showed that the application of 75% and 50% FC of SW decreased FDW significantly as compared to that of control by 10% and 32%, respectively. The results also showed that the application of 2.5, 5, and 7.5 L 1000^−1^ of the organic fertilizer significantly increased FDW by 13%, 20%, and 20%, respectively, as compared to that of the control (Fig 2b). The highest mean value of FDW was 18.22 g pot^-1^ which obtained with the application of 7.5 L 1000^−1^ of the organic fertilizer. The results showed that the highest and lowest FDW were related to W100Z7.5 and W50Z7.5 treatments, respectively. Application of the organic fertilizer at two SW levels of 50 and 75% FC (except for the W50Z7.5 treatment) significantly increased the FDW as compared to that of control.

#### Ascorbic acid (vitamin C) and total titratable acids (citric acid)

**Fig 3:**
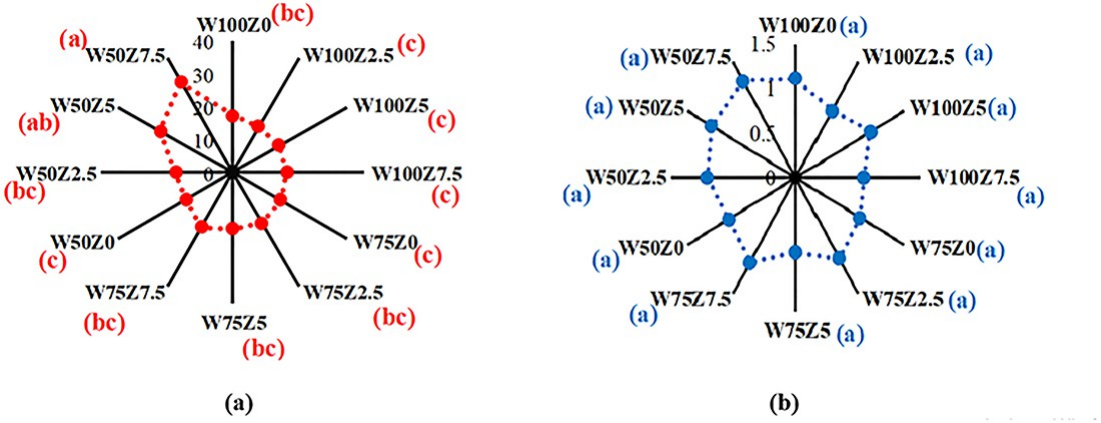
Effect of different levels of Zargreen liquid organic fertilizer (Z) and soil water, drought stress (W) on (a) vitamin C (mg 100 mL^-1^) and (b) citric acid (%) (the distance between points and the origin represents the numerical value under treatments).

Results of ANOVA showed that both foliar application of the organic fertilizer and soil water had a significant effect on the vitamin C concentration of tomato fruit. The result of the mean comparison revealed that the mean value of vitamin C increased by 7% and 37%, under 50% and 75% FC levels of water stress as compared to that of control, respectively, although only the 37% increase was statistically significant. The results also indicated that the application of 5 and 7.5 L 1000^−1^ of the studied organic fertilizer significantly increased the value of vitamin C in tomato fruit compared to that of the control by 16 and 33%, respectively (Fig 3a). The highest mean value of vitamin C (22.4 mg 100 mL^-1^) was obtained by adding 7.5 L 1000^−1^ of the organic fertilizer.

The maximum content of vitamin C (31.68 mg 100 mL^-1^) was obtained in W50Z7.5 followed by W50Z5 (25.39 mg 100 mL^-1^), while the minimum value of vitamin C (15.99 mg 100 mL^-1^) corresponded to the W100Z2.5. It can be concluded that at the highest level of drought stress (50% FC) and the most amount of fertilizer sprayed (7.5 L 1000^−1^), the greatest vitamin C was recorded.

Results revealed that the application of 50% FC increased the mean value of citric acid concentration in tomato fruit as compared to that of the control, but it was not statistically significant. The results also showed that foliar application of the studied organic fertilizer increased the citric acid concentration, but the increase was not statistically significant compared to that of the control (Fig 3b). The highest citric acid concentration (1.05%) was obtained in response to the application of 7.5 L 1000^−1^ of the organic fertilizer.

#### Calcium, potassium, and sodium concentration of fruit

According to the results of variance analysis, both the organic fertilizer and soil water treatments had significant effects on the fruit Ca concentration. The comparison of mean values also showed that the use of 75 and 50% FC levels significantly decreased the mean value of fruit Ca concentration by 18 and 25% as compared to that of control, respectively.

**Fig 4:**
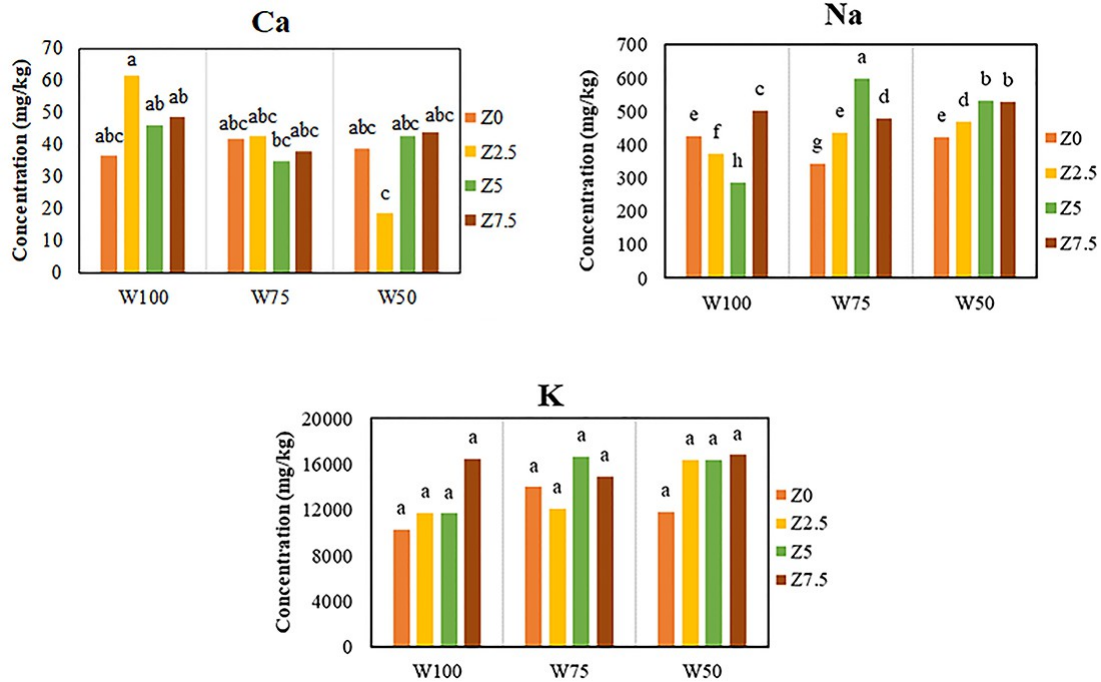
Effect of foliar application of different levels of Zargreen liquid organic fertilizer (Z) and soil water, drought stress (W) levels on the fruit Ca, K, and Na concentrations.

Results also showed that the application of 2.5, 5, and 7.5 L 1000^−1^ of the organic fertilizer increased the fruit Ca concentration compared to the control by 5, 6, and 11%, respectively, although the 5 and 6% increases were not statistically significant (Fig 4). The highest fruit Ca concentration was 43.22 mg kg^-1^ which was obtained when 7.5 L 1000^−1^ of the organic fertilizer was applied. The highest and lowest fruit Ca concentrations were obtained in W100Z2.5 and W50Z2.5 treatments, respectively, although the differences were not statistically significant as compared to that of the control.

The comparison of means value showed that the application of 75 and 50% FC levels as compared to that of control increased fruit K concentration by 15 and 22%, respectively.

Results also showed that the application of 2.5, 5, and 7.5 L 1000^−1^ of the organic fertilizer significantly increased the fruit K concentration as compared to that of control by 11, 24 and 33%, respectively (Fig 4). The highest mean value of fruit K concentration was 16070 mg kg^-1^ in the plants subjected to foliar application of 7.5 L 1000^−1^ of the organic fertilizer. Application of the organic fertilizer at all levels of soil water increased the fruit K concentration, although this increase was not statistically significant as compared to that of control.

The results of variance analysis showed that foliar application of the organic fertilizer as well as soil water had a significant effect on the fruit Na concentration. The comparison of mean values showed that application of 75% and 50% FC significantly increased fruit Na concentration by 17% and 23% as compared to that of control, respectively. The results also indicated that the application of 2.5, 5, and 7.5 L 1000^−1^ of the organic fertilizer significantly increased fruit Na concentration as compared to that of control by 7, 19, and 27%, respectively (Fig 4). The highest mean value of fruit Na concentration was 504 mg kg^-1^ which was obtained when 7.5 L 1000^−1^ of the organic fertilizer was applied. In general, the results revealed that the application of the organic fertilizer at different levels of water deficit (soil water), especially at the highest level (W50), increased significantly the fruit Na content.

#### Iron, manganese, copper, and zinc concentration of fruit

**Fig 5:**
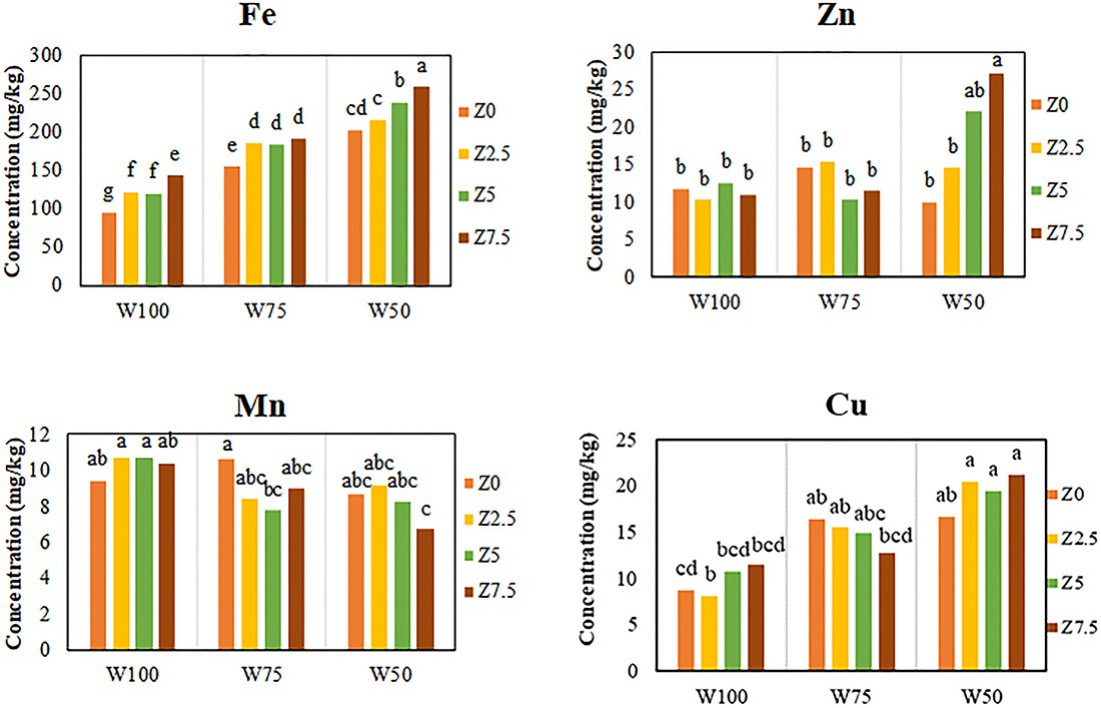
Effect of foliar application of different levels of Zargreen liquid organic fertilizer (Z) and soil water, drought stress (W) levels on fruit Fe, Mn, Zn, and Cu concentrations of tomato.

Analysis of variance showed that foliar application of the organic fertilizer and soil water had significant effects on the fruit Fe concentration. Results of mean comparison also showed that under 75 and 50% FC soil water conditions significantly increased fruit Fe concentration by 49 and 91% as compared to that of control, respectively. In addition, application of 2.5, 5, and 7.5 L 1000^−1^ of the organic fertilizer significantly increased fruit Fe concentration by 16, 20, and 31% as compared to that of control, respectively (Fig 5). The highest mean value of fruit Fe concentration (198 mg kg^-1^) was obtained after application of 7.5 L 1000^−1^ of the organic fertilizer. Results showed that addition of the organic fertilizer for all applied levels of soil water significantly increased the fruit Fe concentration as compared to that of the control. The highest and the lowest Fe concentrations were observed in response to applying the W50Z7.5 and W100Z0 treatments, respectively.

According to the analysis of variance, both applied treatments (the organic fertilizer and soil water) had significant effects on the fruit Mn concentration. Results showed that applying soil water levels of 75% and 50% FC significantly decreased the mean fruit Mn concentration by 13% and 20% as compared to that of the control, respectively. Findings also showed that application of 2.5, 5, and 7.5 L 1000^−1^ of the organic fertilizer decreased the fruit Mn concentration by 1, 7, and 9%, respectively (*P* > 0.05). The highest mean value of fruit Mn concentration was 9.55 mg kg^-1^ which was obtained in the control. The interaction of soil water and the organic fertilizer did not show a clear trend regarding the fruit Mn concentration.

Analysis of variance showed that foliar application of the organic fertilizer as well as soil water had significant effects on fruit Zn concentration. Results showed that under soil water levels of 75 and 50% FC, fruit Zn concentration increased significantly by 11 and 63%, compared to that of control, respectively. Findings indicated that application of 2.5, 5, and 7.5 L 1000^−1^ of the studied organic fertilizer significantly increased the fruit Zn concentration as compared to that of control by 9, 12, and 24%, respectively. However, the 9% increase was not statistically significant. The highest mean value of fruit Zn concentration (16.53 mg kg^-1^) was obtained with the application of 7.5 L 1000^−1^ of the studied organic fertilizer which was observed when the highest level of drought stress and the highest level of organic fertilizer (W50Z7.5) was applied.

The results of variance analysis showed that foliar application of the organic fertilizer as well as soil water had significant effects on fruit Cu concentration. Mean comparison indicated that under soil water levels of 75% and 50% FC, fruit Cu concentration increased significantly by 53% and 99% as compared to that of control, respectively. In addition, the application of 2.5, 5, and 7.5 L 1000^−1^ of the studied organic fertilizer increased the Cu concentration of tomato fruit by 5, 8, and 9%, respectively (*P* > 0.05). The highest mean value of Cu concentration in tomato fruit was 15.15 mg kg^-1^ which was obtained with applying 7.5 L 1000^−1^ of the organic fertilizer. The interaction effect of soil water and the organic fertilizer did not show a clear trend regarding fruit Cu concentration.

### Principal component analysis (PCA)

**Fig 6:**
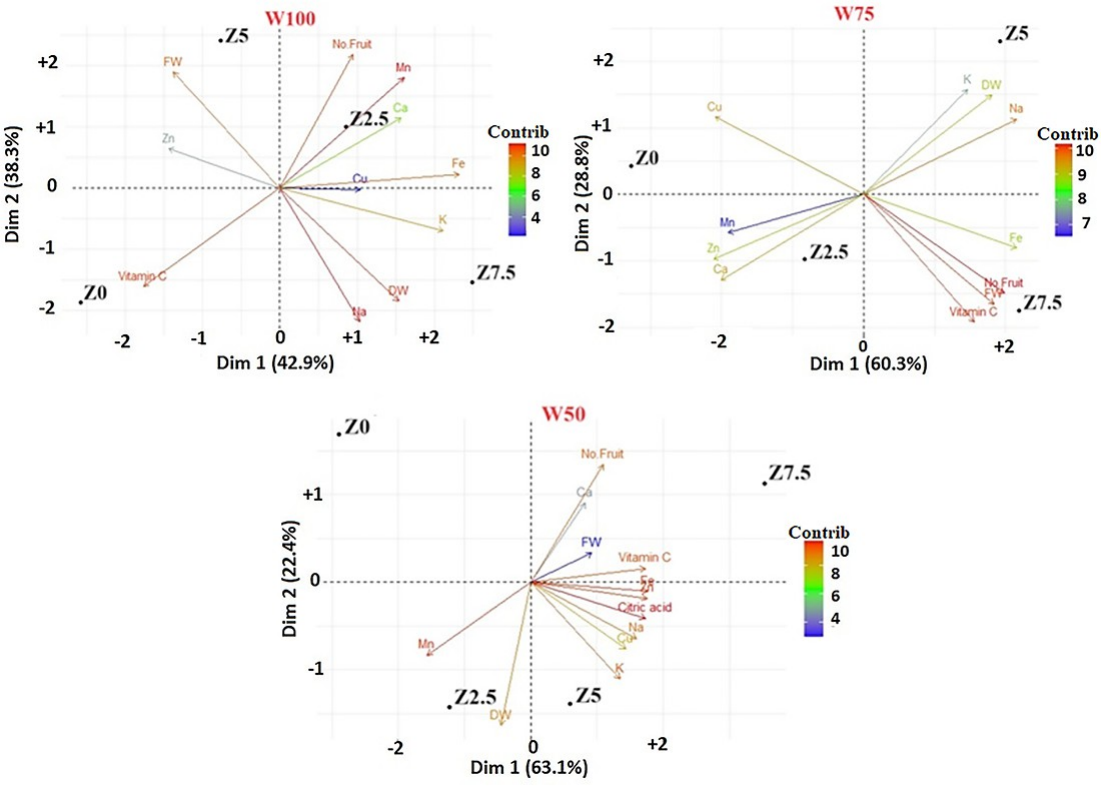
Biplot diagram derived from the first and second-factor components for various treatments.

Fig 6 indicates the contribution of various levels of the applied organic fertilizer on tomato yield and growth parameters under soil water (drought stress) levels, as presented by biplot. The results of the biplot model using the different treatments representing 42.9% and 38.3%, 60.3% and 28.8%, and 63.1% and 22.4% of the variance contribution to the first two principal components for the PC1 and PC2, under W100, W75, and W50 soil water treatments, respectively. The length and angle of principal vectors (variables) indicate variance and covariance, respectively. Vitamin C, number of fruits, FDW, fruit Na and Mn concentrations under W100, vitamin C, number of fruits, and FFW under W75, citric acid, and fruit Fe, Mn, and Zn concentrations under W50 were found with higher magnitude due to their longest vector length among all of the studied traits; therefore, they contributed most in overall variation.

All traits with smaller or closer vector angles with each other show a positive relationship with each other (i.e., angles of < 90° and > 90° 90°, indicating positively and negatively correlated variables, respectively, and angles of 90° showing independent variables). In the present study, without application of the organic fertilizer, no correlation was observed between the measured properties in any of the soil water treatments. Meanwhile, under the condition of using different levels of the organic fertilizer positive correlations between the characteristics were observed. In other words, there were positive correlations between Na concentration and FDW under W100Z7.5; between number of fruits and Mn concentration under W100Z2.5; between vitamin C, FFW, and number of fruits under W75Z7.5; between Zn, Mn, and Ca concentrations under W75Z2.5; and between FDW, K, and Na concentrations with each other under W75Z5 treatment. Furthermore, there were positive correlations between the concentrations of Fe, Zn, Na, Cu, K, and citric acid of tomato fruit under the application of W50Z5 treatment, and between vitamin C, citric acid, FFW, number of fruits, and Fe concentration under W75Z7.5 and between Zn, Mn, and Ca concentrations under W75Z2.5 treatments.

The best treatments can be selected with increased PC1 and decreased PC2 for different soil water conditions. The W100Z7.5, W75Z7.5, and W50Z5 treatments with the higher PC1 and the lower PC2, also exhibited higher scores for FDW, Na, and K concentrations under W100; vitamin C, number of fruits, FFW, and Fe concentration under W75; and citric acid, Fe, Zn, Na, K, and Cu concentrations under W50. Having high PC1 and PC2 were obtained with W100Z2.5, W75Z5, and W50Z7.5, and scored higher values for the number of fruits, Mn, Ca, and Fe concentrations in W100; FDW, K, and Na concentrations in W75; the number of fruits, FFW, Ca, and vitamin C in W50. The W100Z0, W75Z2.5, and W50Z2.5 the lower PC1 and PC2, with higher scores for vitamin C in W100; Mn, Zn, and Ca concentration in W75; Mn and FDW in W50. The lower PC1 and the higher PC2 were observed with W100Z5, W75Z0, and W50Z0, scoring higher values for FFW and Zn concentration under W100; and Cu concentration under W75 which is not recommended.

### Relationships between the growth traits of tomato

**Fig 7:**
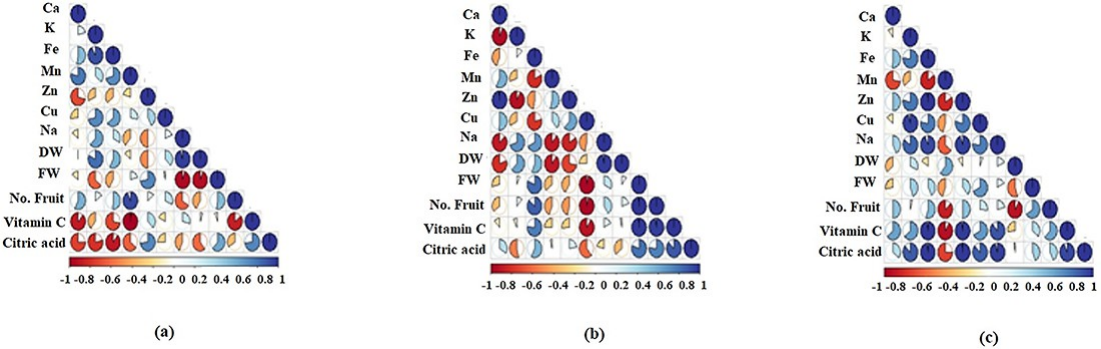
Correlation matrix between different plant growth traits under various water stress, (a), W100; (b), W75; (c), W50.

According to Fig 7a, under W100 soil water treatment, a strong positive correlation was obtained between FDW and Na concentration (r = 0.96); between the number of fruits and Mn concentration (r = 0.93); and between Fe and K concentrations (r = 0.91). The FFW content was negatively correlated with Na concentration (r = -0.95) and FDW (r = -0.94). Vitamin C was negatively correlated with Mn concentration (r = -0.98), Ca concentration (r = -0.88), and number of fruits (r = -0.86). There was a negative correlation (r = -0.89) between citric acid and Fe concentration. Based on Fig 7b, under W75, a positive correlation was observed between Ca and Zn concentration (r = 0.99), vitamin C and FFW (r = 0.99), vitamin C and number of fruits (r = 0.97), FFW and number of fruits (r = 0.99). There was a negative correlation between Cu concentration with FFW (r = -0.96), number of fruits (r = -0.97), and vitamin C (r = -0.91), and between Na and Ca and Mn concentrations (r = -0.88 and -0.89, respectively), FDW with Mn and Ca concentrations (r = -0.90 and -0.80, respectively). Fig 7c indicated that under W50, vitamin C was positively correlated with Fe (r = 0.99), Zn (r = 0.98), citric acid (r = 0.93), and Na concentrations (r = 0.89). Citric acid content was positively correlated with Fe (r = 0.98), Zn (r = 0.99), K (r = 0.88), and Na concentrations (r = 0.96). There were positive correlations between Na and Zn (r = 0.95), Cu and K (r = 0.96), Zn and Fe (r = 0.99), and Na and Fe (r = 0.94) concentrations. Mn concentration negatively correlated with citric acid (r = -0.75), Zn (r = -0.83), vitamin C (r = -0.92), and number of fruits (r = -0.86). Furthermore, negative correlations were observed between the number of fruits and FDW (r = -0.92), and Mn and Fe concentrations (r = -0.86).

## Discussion

Drought, as one of the most detrimental environmental factors, limits the plant growth and yield. Organic fertilizer addition is proposed as an effective management strategy to mitigate the adverse effects of drought [1, 5, 6, 42, 43]. Results showed that water deficit decreased the number of fruits while the organic fertilizer increased it. According to the results, the applied organic fertilizer at the maximum level decreased the negative impact of drought on plants by enhancing the number of fruits per plant. As explained by Agbna et al. [44] and Ghezzehei et al. [45], the application of biochar, as an organic compound, significantly increased the number of fruits per plant by supplying some essential nutrients and storing them in the matrix and increasing nutrient availability. Usman et al. [46] showed that the maximum number of fruits was obtained at 70% FC and the addition of 0.2% biochar; while its minimum corresponded to plants without biochar application at the water deficit level of 50% FC. Hossain [47] reported that the highest number of fruits per tomato plant was observed in treatment with one-day interval irrigation and application of 5 t ha^-1^ poultry manure, while, the lowest number was found in control (no fertilizer with two-day interval irrigation).

Findings indicated that fresh (FFW) and dry (FDW) weight of tomatoes decreased under drought stress; however, application of the Zargreen organic fertilizer increased these parameters. The fruit weight is the product of photosynthesis, and the uptake of water, nutrients, and chlorophyll contents during photosynthesis was negatively affected by water deficits. If the crop evapotranspiration overcomes the water uptake, water supply by roots will be lessened, which causes an internal water deficiency affecting photosynthesis, resulting in intercellular volume and cell size reduction, as well as reduced accumulation of fruit water content and consequently a decrease in fruit weight [27, 48]. Alteration in fruit weight can be attributed to water stress, in particular the growth stage, intensity, and the period at which the stress occurs [49]. Keabetswe et al. [50] observed that tomato fruit weight decreased significantly due to water shortage, while, biochar application by improving water holding capacity, had a positive effect on fruit quality which is in accordance with our findings. Aires et al. [51] showed that the positive effects of salicylic acid foliar application in tomatoes grown under drought stress were due to the effect of this compound on gas exchanges, especially CO_2_ accumulation and carboxylation performance in the plant. Basiouny et al. [52] showed that as water stress levels increased the concentration of abscisic acid and ethylene increased; whereas, weight of the fruits and the amount of free carbohydrates decreased significantly. Behbodi et al. [53] reported that the application of chitosan nanoparticles reduced the negative effects of drought stress, as a result, barley plant growth and performance increased. Haghigi and Najafi [54] showed that drought stress reduced the fresh and dry weight of tomatoes, while the addition of humic acid in the same conditions improved the relative water content of the tissue, the fresh and dry weight of the shoots. Mubashir et al. [55] also showed that the weight of fruit, fresh weight of shoots, and roots of tomato under moisture stress (60% FC) decreased by 18.3%, 31.8%, and 14.6%, respectively, compared to the control. The reduced water supply significantly affects fruit size, specifically the weight [56]. Haghigi and Najafi [54] stated that the application of humic acid treatments under moisture-stress conditions had no significant effect on the fresh and dry weight of tomato fruit. Usman et al. [46] indicated that the highest fruit dry weight of tomato was obtained with the application of 0.1 and 0.2% of biochar with 70% FC drought stress. They showed that the lowest dry weight was observed without biochar or with 0.1% biochar at the same level of drought stress (50% FC). Based on the results of Konozy et al. [57], in many instances, drought stress decreased plant protein content, resulting in a reduction of cell wall enzymes such as β-glucosidase, β-mannosidase, and α-galactosidase, finally in fruit softening. Biochar application decreased the adverse effects of drought stress by improving protein contents, which increased the fruit quality by storing water and nutrients for a longer time. Plant regulators create plant tolerance to drought stress by modifying biochemical and physiological processes, for example, keeping stomata open. Abscisic acid is one of the plant regulators in response to moisture deficiency. This combination causes the stomata to close through secondary messengers such as active oxygen, nitric oxide, and calcium. The application of salicylic acid reduces the amount of abscisic acid and ethylene, as a result of which stomatal conductance and photosynthesis increase in drought stress conditions [58]. It has been reported that the maximum fruit yield of tomatoes was observed as a result of poultry manure at 5 t ha^-1^ with two-day interval irrigation [59].

According to the results, at the highest level of water deficit (50% FC) and the highest level of the organic fertilizer sprayed (7.5 L 1000^−1^), the greatest vitamin C concentration was recorded. This result is in agreement with Chen et al. [60], who stated that vitamin C was positively influenced by water stress in tomatoes. Nahar and Gretzmacher [61] also indicated that the concentration of ascorbic acid in fruit increased with water stress and the highest content was observed at 40% FC conditions. In other words, they reported that citric acid content significantly increased by 69% as compared to that of control when plants were subjected to the highest level of drought stress (40% FC). Antonio et al. [62] noted that the vitamin C content in vegetables is influenced by many factors, among them the maturity level, cultivar and plant nutrition, and water content are the most influential ones. Keabetswe et al. [50] showed that vitamin C, as a fruit quality attribute, increased under water stress at various growth stages. Vitamin C synthesis is associated with carbohydrate metabolism and starts with glucose. The higher sugar accumulation in the plant with lower water content promoted vitamin C synthesis during fruit ripening. In addition, water deficit reduced leaf area index and thus increased light intensity and duration for fruit [27], which is appropriate for the accumulation of vitamin C [63]. According to our findings, the maximum fruit Fe, Zn, and Cu concentrations were obtained under the highest level of applied water deficit (W50) with application of the greatest amounts of the organic fertilizer (7.5 L 1000^−1^). Our results were in agreement with the findings of Najafi-Ghiri et al. [64]. The results also were in agreement with Nahar and Gretzmacher [61], who reported that Ca and Mg concentrations of tomatoes decreased with increasing water stress. They also stated that the highest and lowest K concentrations in tomatoes were observed at 100% FC (control) and 40% FC, respectively. Increased plant growth and nutrient content due to the application of the organic fertilizer have been attributed to its several beneficial attributes such as the presence of high mineral content and higher availability of soil water to plant roots due to its probable positive impacts on the influential soil physical attributes such as total porosity, pore size distribution, aggregation and aggregate stability, and water holding capacity of the soil. The mentioned positive impacts of organic compounds on water availability- related soil attributes have been reported by the other investigators [1, 65, 66].

## Conclusion

Drought stress is the most limiting factor for plant growth in arid and semiarid regions. This is the first study to investigate the potential of Zargreen liquid organic fertilizer to mitigate the adverse effects of water deficit on tomato growth, yield, and its fruit chemical composition. In the present study, different levels of the organic fertilizer were foliar sprayed on tomato plants grown under different levels of soil water (drought stress) conditions. In general, findings indicated that water deficit adversely affected and decreased fruit fresh and dry weights of tomatoes and their Ca concentration. Whereas, K, Na, Cu, Fe, and Zn concentrations and vitamin C content of tomato fruit increased under drought conditions. The quantity and quality of tomato plants treated with the organic fertilizer were better than that of the control with respect to vitamin C content, fresh and dry weights, and nutritional status. Foliar spraying of the organic fertilizer was effective in improving the quantity and quality attributes of tomato fruit under water deficit conditions. It can be concluded that applying organic fertilizer with a high amount of amino acid contents and essential nutrient elements, can improve the amount of chlorophyll and cell metabolism and consequently the quantity and quality attributes of plants under drought conditions.
